# Altitudinal and Seasonal Variation in *Drosophila* Species on Mount Japfu of Nagaland, a Sub-Himalayan Hilly State of India

**DOI:** 10.1673/031.013.11701

**Published:** 2013-10-26

**Authors:** Bovito Achumi, Shridhar N. Hegde, Pardeshi Lal, Sarat Chandra Yenisetti

**Affiliations:** 1 Department of Zoology, Nagaland University (Central), Lumami- 798627, Nagaland, India; 2 Department of studies in Zoology, Manasagangotri, University of Mysore, Mysore- 570006, Karnataka, India

**Keywords:** cluster analysis, occurrence constancy method, Simpson's diversity index

## Abstract

*Drosophila* (L.) (Diptera: Drosophilidae) has richly contributed to the understanding of patterns of inheritance, variation, speciation, and evolution. *Drosophila*, with its cosmopolitan nature and complexities in species compositions, is an excellent model for studying the eco-distributional patterns of various species. This study analyzed the altitudinal and seasonal variation in *Drosophila* species of Mount Japfu in Nagaland, a sub-Himalayan hilly state of northeast India, over the course of one year. A total of 4,680 *Drosophila* flies belonging to 19 species of 4 subgenera were collected at altitudes of 1500, 1800, 2100, 2400, and 2700 m a.s.l. The subgenus *Sophophora* Sturtevant was predominant, with 10 species, followed by subgenus *Drosophila*, with 4 species. Subgenus *Dorsilopha* and subgenus *Scaptodrosophila* were represented by 1 species each. The remaining 3 species were not identified. Cluster analysis and constancy methods were used to analyze the species occurrence qualitatively. Altitudinal changes in the population densities and relative abundances of the different species at different seasons were also studied. The diversity of the *Drosophila* community was assessed by applying Simpson's diversity index. At 1800 m a.s.l., the Simpson's index was low (0.09301), suggesting high *Drosophila* diversity at this altitude. The density of *Drosophila* changed significantly during different seasons (F = 26.72; df = 2; *p* < 0.0001). The results suggest the distributional pattern of a species or related group of species was uneven in space and time.

## Introduction

The fruit fly *Drosophila* (L.) (Diptera: Drosophilidae) has richly contributed to the understanding of patterns of inheritance, variation, speciation, and evolution. Genus *Drosophila*, with its cosmopolitan nature and complexities in species compositions, is an excellent model for studying the ecodistributional patterns of various species (Carson 1965). Systematic study concerning the variations in the species compositions and the patterns of distribution of various members of the genus *Drosophila* in different geographical regions of the world will enable understanding of the principles underlying adaptive radiation and certain mechanisms involved in speciation (Muniyappa 1981).

Significant progress has been made in the fields of taxonomy and systematics of the family Drosophilidae in India. This family is composed of more than 3,500 species throughout the world (Bachli 1998). About 200 species belonging to 20 genera have been reported from different parts of India. However, very little is known regarding the *Drosophila* fauna of the northeastern region of the Indian subcontinent. This region is one of the richest repositories of biodiversity in the world, with its diverse climatic conditions, variable altitudes, deep valleys, luxuriant flora, running streams and moist surroundings. So, it provides an ideal location for the colonization of several *Drosophila* species (Singh and Gupta 1977; Dwivedi and Gupta 1979; Gupta and Singh 1979; Singh and Gupta 1980; Singh 1987). This region includes eight hill states, namely Assam, Arunachal Paradesh, Manipur, Meghalaya, Mizoram, Nagaland, Sikkim, and Tripura. A preliminary survey on Drosophilids of Dimapur, Medziphema, and Kohima of Nagaland state was conducted (Singh 1987). A preliminary report was published by Yenisetti et al. (2002) on Drosophilids of Mokokchung town. However, no systematic comprehensive study has been done on Drosophilids of Nagaland, a sub-Himalayan hilly state in the northeast region of India. It is possible that new *Drosophila* species can be identified from this region. *Drosophila* is a pollinator for economically important plants, such as *Ceropegia* (Chaturvedi 2006). It is possible that novel *Drosophila* pollinators for other economically important plants can be identified in these subtropical rain forests.

The ecological and biological diversity of an ecosystem determines the presence or absence of a species in an ecological niche. Apart from physical and biotic factors, the topography and season also affect animal distribution. As elevation is one of the important aspects of topography, it is important to look at animal distribution from that perspective. Efforts have been made to collect *Drosophila* from different altitudes, but these data were not considered with an ecological perspective (Reddy and Krishnamurthy 1977). According to Reddy and Krishnamurthy (1977), physical and biotic factors are the sole determinants of animal distribution. This idea logically denotes that elevation and season have no influence on animal distribution. In the present study, our goal was to determine if elevation affects distribution.

According to Gause's competitive exclusion theory, two related species competing for the same resources cannot co-exist together in the same ecological niche (Gause 1934). However, laboratory experiments questioned the validity of this principle (Ayala 1969). The presence of taxonomically or phylogenetically related species in an ecological niche indicates their coexistence, and absence of such related species infers competitive exclusion (Guruprasad et al. 2010). Our study sought to understand whether taxonomically or phylogenitically related *Drosophila* species coexist in nature. Our study also has been undertaken to understand the altitudinal and seasonal variation of *Drosophila* species on Mount Japfu, which is situated 15 km from Kohima, the capital of the sub-Himalayan hilly state Nagaland, India.

## Materials and Methods

The altitudinal and seasonal fluctuation in *Drosophila* fauna was studied in five different wild localities of Mount Japfu, which has a peak altitude of about 3015.60 m a.s.l. Its slopes are covered with thick vegetation. The selected collection spots were located at 25° 11′ N latitude and 94° 55′ E longitude. Monthly collections of flies were made at the altitudes of 1500, 1800, 2100, 2400, and 2700 m a.s.l. from April 2010 to March 2011. Both bottle trapping and net sweeping methods were used. For bottle trapping, milk bottles of 200 mL capacity containing a smashed ripe banana sprayed with yeast were tied to the twigs underneath small bushes at the height of 1–1.5 m above the ground. Fifteen traps were kept at each site. After 2 days, the mouth of each bottle was plugged with cotton and removed from the bushes. The flies that were attracted by the bait and collected in the bottles were transferred to fresh bottles containing wheat cream agar medium. The medium was prepared by adding 100 g of sugar (jaggery) to 500 mL of water and boiling it. Then, 500 mL of water, 100 g of wheat powder (soji), and 8 g of agar-agar were added to the boiling sugar water mixture. When the medium turned sticky, 7.5 mL propionic acid (anti-fungal agent) was added while continuously stirring the medium.

Net sweeping was done on rotting fruits (crushed banana spread beneath shaded areas of bushes 1 day before collection). After each sweep, collected flies were transferred to bottles containing freshly prepared wheat cream agar medium.

The flies were then brought to the laboratory, isolated, identified, and sexed. Categorization of the collected *Drosophila* flies was made to respective taxonomic groups by employing the parameters as suggested by Sturtevant (1921), Patterson and Stone (1952), Throckmorton (1962), and Bock (1971). To study seasonal variation, the entire year was divided into three seasons: pre-monsoon, extending from January through March, monsoon, from April through September, and post-monsoon, from October through December.

### Flora of the collection sites:

Following is a brief description of the flora available in each of the collection spots.

Flora at 1500 m a.s.l.: maibau, *Alnus nepalensis* (Don) (Fagales: Betulaceae); beggarticks, *Bidens* spp (Asterales: Asteraceae); bittervine, *Makania* spp; sow thistles, *Sonchus* spp; butterfly bush, *Buddleja* spp (Lamiales: Scrophulariaceae); brahmi booti, *Centella asiatica* (L.) (Apiales: Apiaceae); sirib large, *Entada pursathea* (Roux) (Fabales: Fabaceae); banana, *Musa* spp (Zingiberales: Musacae); carrion flowers, *Smilax* spp (Liliales: Smilacaceae); pinyin, *Stemona* spp (Pandanales: Stemonaceae); currant tomato, *Solanum* spp (Solanales: Solanaceae); marda, *Termenalia elliptice* (Wright & Arn) (Myrtales: Combretaceae); etc.

Flora at 1800 m a.s.l.: jackfruit, *Artocarpus hetrophyllus* (Lam) (Rosales: Moraceae); yellow Himalayan raspberry, *Rubus* spp (Rosaceae); wormwood, *Artemisisia vulgaris *(L.) (Asterales: Asteraceae); beggar-ticks, *Bidens* spp; bamboo, *Bambusa* spp, (Poales: Poaceae); black musale, *Curculigo* spp (Asparagales: Hypoxidaceae); timburni, *Diospynum* spp, (Ericales: Ebenaceae); deereye beans, *Mucuna perita* (Adans) (Fabales: Fabaceae); tapioca-root, Maninot utilissema (Crantz) (Malpighiales: Euphorbiaceae); *Smilax* spp; khasi pine, *Pinus insularies* (Gordon) (Pinales: Pinaceae); knotwood, *Polygonum* spp (Caryophyllales: Polygonaceae); etc. Flora at 2100 m a.s.l.: *A. nepalensis* (Don); khang, *Acacia pinnata* (Miler) (Fabales: Fabaceae); thickhead, *Crossocephalum* spp (Asterales: Asteraceae); Himalayan nettle, *Girardinia heterophylla* (Vahl) (Rosales: Urticaceae); *Rubus* spp; blady grass, *Imperata cylindrica* (Brauv) (Poales: Poaceae); *Musa* spp; etc.

Flora at 2400 m a.s.l.: bologi, *Crossocephalum* spp (Asterales: Asteraceae); *A. vulgaris*; white weed, *Ageratum conyzoids* (L.); thoroughworts, *Eupatorium* spp; *Bidens* spp; blueberry ash, *Elaeocarpus* spp (Oxalidales: Elaeocarpaceae); shaking brake, *Pteris* spp (Polypodiales: Pteridaceae); *I. cylindrica*; *C. asiatica*; *P. insularies*; knotwood, *Polygonum* spp; cowitch, *Mucuna pruriens* (L.) (Fabales: Fabaceae); etc.

Flora at 2700 m a.s.l.: *Crossocephalum* spp; *Curculigo* spp; *I. cylindrica*; kamraj, *Helminthostachys zeylanica* (L.) (Ophioglossales: Ophioglossaceae); *Polygonum* spp; *Smilax* spp; timburni, *Dryopteris* spp (Ericales: Ebenaceae); rhododendron, *Rhododendron* spp (Ericaceae); etc.

### Data analysis

The relationship between the abundance, richness, and diversity of all groups of flies collected throughout the year was calculated by Simpson's diversity index (Simpson 1949). Simpson's diversity index (D) measures the probability that 2 individuals randomly selected from a sample will belong to the same species, and was calculated using the following formula:

Where n = the total number of organisms of a particular species, and N = the total number of 
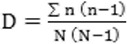
 organisms of all populations.

In order to verify the qualitative distribution of different species, the occurrence constancy method (Dijoz 1983) was used. The constancy value (C) was obtained by dividing the number of collections in which one species occurred by the total number of collections, and then multiplying that result by 100. Based on constancy value, the species collected were grouped as constants when C ≥ 50, accessory species when C ≥ 25 and < 50, and accidental species when C < 25. Species that occurred in only one area were considered exclusive.

To understand the difference in seasonal variation of *Drosophila* flies at Mount Japfu, oneway analysis of variance was performed using GraphPad Prism5 software (www.graphpad.com).

Cluster analysis was performed using WinSTAT software (www.winstat.com) to design, analyze, and compare different *Drosophila* populations, as described by Giri et al. (2007). In the cluster study, Euclidean distance was chosen to measure the similarity between different species, and Ward's strategy (Giri et al. 2007) was performed to unite two clusters.

**Table 1.**
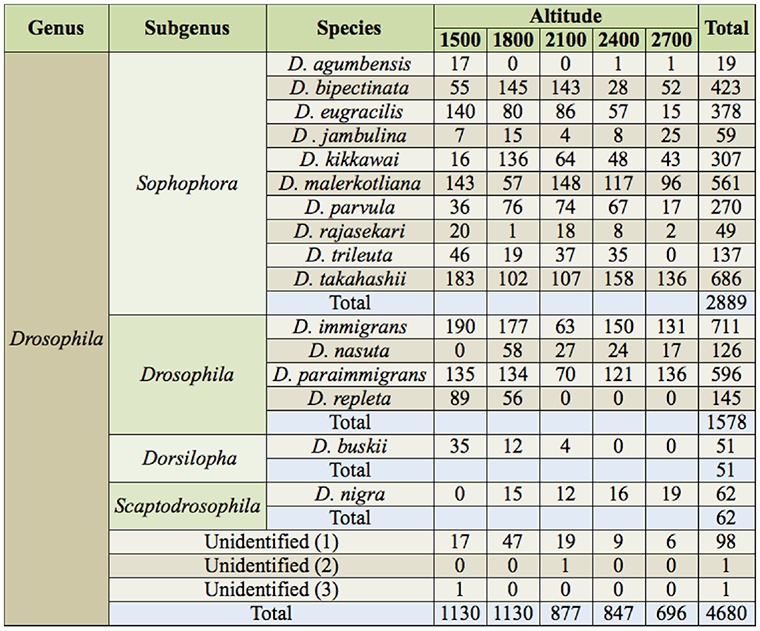
The list of species of *Drosophila* collected, and the number of *Drosophila* collected at different altitudes (m a.s.l.) of Mount Japfu from April 2010 to March 2011.

## Results

The list of *Drosophila* species collected at different altitudes of Mount Japfu from April 2010 through March 2011 and their taxonomic position are given in Table 1. A total of 19 species were collected, including 16 species of *Drosophila* belonging to 4 subgenera (*Sophophora*, *Drosophila, Dorsilopha* and *Scaptodrosophila*). The remaining 3 species were not identified. Pooled data on monthly collections of *Drosophila* yielded a total of 4680 individuals. Out of these, 2889 individuals (61.73%) belonged to 10 species of subgenus *Sophophora*, 1578 (33.71%) individuals belonged to 4 species of the subgenus *Drosophila* 100 (2.13%), 3 were unidentified, 62 (1.32%) individuals belonged to 1 species of subgenus *Scaptodrosophila*, and 51 (1.07%) belonged to 1 species of subgenus *Dorsilopha*.

The value of the Simpson's index, indicating the abundance, richness, and diversity of *Drosophila* flies at different altitudes, is given in (Table 3). At the lowest altitude (1500 m a.s.l.), the Simpson's index was 0.10903, and at the highest altitude (2700 m) it was 0.141355.

**Figure 1.**
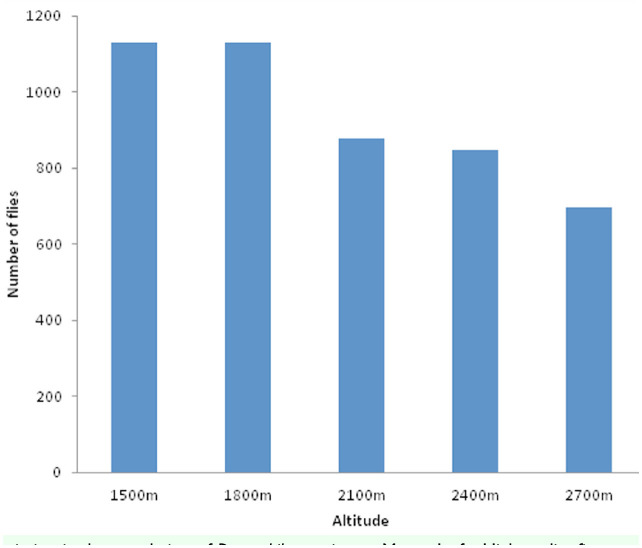
Altitudinal variation in the population of *Drosophila* species on Mount Japfu. High quality figures are available online.

**Figure 2.**
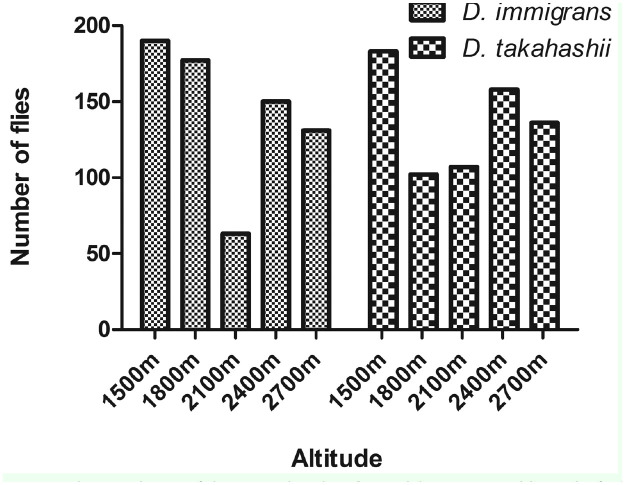
Altitudinal variation in the population of the most abundant *Drosophila* species on Mount Japfu. High quality figures are available online.

The altitudinal variation of the *Drosophila* population is depicted in (Figure 1). The number of *Drosophila* flies decreased with increasing altitude. The altitudinal variations of the most abundant *Drosophila* species are shown in (Figure 2).

**Figure 3.**
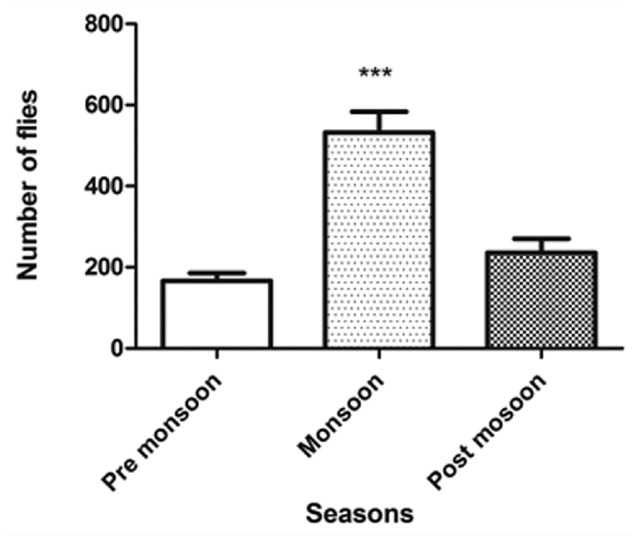
Seasonal variation in *Drosophila* flies collected from Mount Japfu (F = 26.72; df = 2; *p* < 0.0001). High quality figures are available online.

The seasonal variation in the population density of *Drosophila* is depicted in Figure 3. The density was low in the pre-monsoon period, increased in the monsoon period, and then decreased in the post-monsoon period. The analysis of variance calculated for premonsoon, monsoon, and post-monsoon seasons showed significant differences among them (F = 26.72; df = 2, *p* < 0.0001).

**Table 2.**
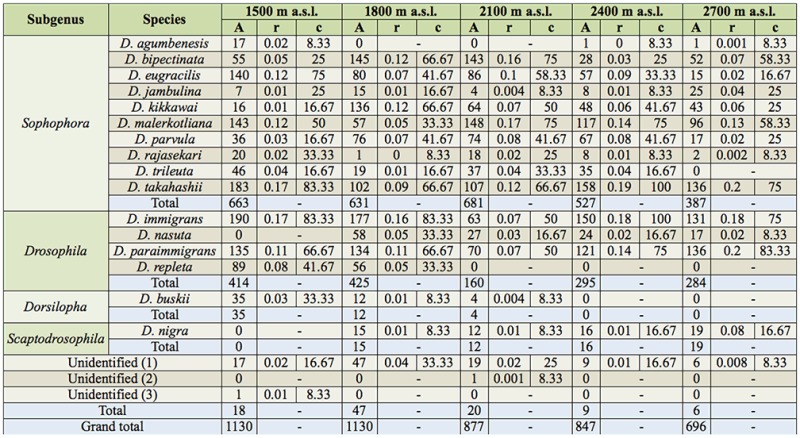
Absolute (A) and relative abundance (r) and constancy values (c) for each species collected at different altitudes of Mount Japfu from April 2010 to March 2011.

**Table 3.**
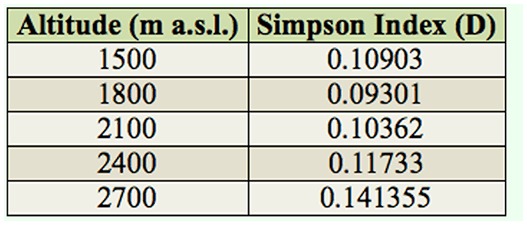
Simpson's diversity index (D) according to the altitude of Mount Japfu.

**Figure 4.**
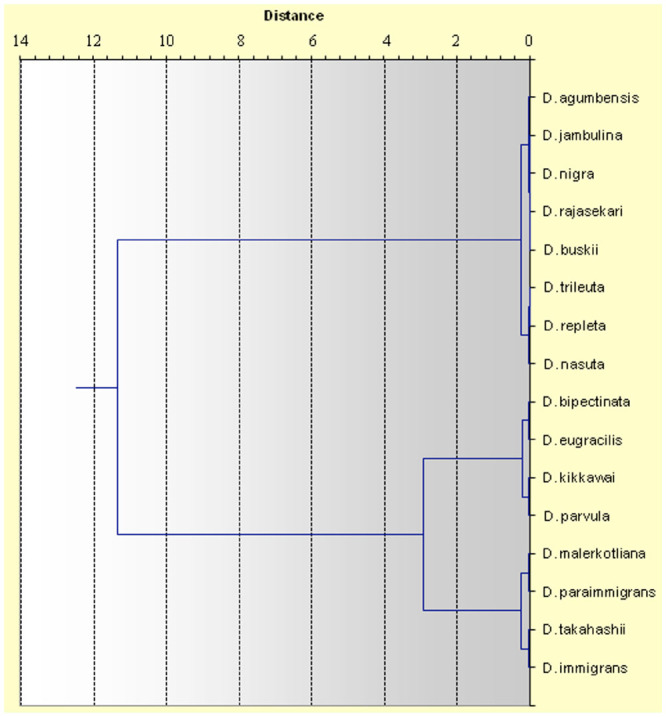
The cluster analysis of *Drosophila* species found on Mount Japfu (dendrogram using Ward's method). High quality figures are available online

The constancy value (C) of all species at all altitudes along with absolute numbers and relative abundance are presented in Table 2. Constant species (C ≥ 50) represented 36.84% of the total collected species (7 out of 19), while 8 species were considered accessory (42.10%), and 4 species were considered accidental (21.05%). Constant species were *Drosophila bipectinata* (Duda) (Diptera: Drosophilidae), *D. eugracilis* (Bock and Wheeler), *D. kikkawai* (Burla), *D. malerkotliana* (Parshad and Paika), *D. takahashii* (Sturtevant), *D. immigrans* and *D. paraimmigrans*; accessory species were *D. jambulina* (Parshad and Paika), *D. parvula* (Bock and Wheeler), *D. rajasekari* (Reddy and Krishnamurthy), *D. trileuta* (Bock and Wheeler), *D. nasuta* (Lamb), *D. replete* (Wollaston), *D. buskii* (Coquilett), and unidentified species (1); accidental species were *D. agumbensis* (Prakash and Reddy), *D. nigra* (Grimshaw), unidentified (2) and unidentified (3). In the cluster analysis (Figure 4), the accidental species stand first in the cluster, followed by the accessory species, and the bottom is occupied by constant species. *D. agumbenesis, D. jambulina, D .rajasekari, D. trileuta* belong to *melanogaster* species group of the subgenus *Sophophora. D. nigra* belongs to subgenus *Scaptodrosophila. D. agumbenesis* and *D. jambulina* belong to *montium* subgroup and *D. bipectinata* belongs to the *ananassae* subgroup. *D. repleta, D. buskii* of the same cluster belongs to subgenus *Drosophila.* In the second cluster *D. eugracilis, D. kikkawai* and *D. parvula* belong to the *melanogaster* species group of the subgenus *Sophophora* and *D. paraimmigrans, D. immigrans* of the same cluster belong to subgenus *Drosophila. D. takahashii* belongs to *melanogaster* species group of subgenus *Sophophora*.

## Discussion

The density of *Drosophila* on Mount Japfu decreased with increasing altitude. The density was high at 1500 and1800 m a.s.l., but was low at 2700 m a.s.l. (Figure 1). The results indicate that the *Drosophila* community was affected by elevation. Wakahama (1961, 1962) has reported similar altitudinal variation in the distribution of *Drosophila* on Mt. Dakesan in Japan. He noticed that the total density of all species decreased with increasing altitude. Reddy and Krishnamurthy (1977) also noticed such altitudinal variation in *Drosophila* populations in the Jogimatii hills of Karnataka. Guruprasad et al. (2010) also observed seasonal and altitudinal variation in *Drosophila* populations of Chamundi Hill in Mysore, Karnataka, India. The reasons behind the observed phenomenon can be attributed to changes that occur as one ascends an altitudinal transect, potentially involving changes in temperature, precipitation, partial pressure of atmospheric gases, atmospheric turbulence and wind speed, and radiation input, including short-wave ultra-violet radiation at different wavelengths (Barry 1992). According to Hodkinson (2005), the above-mentioned changes are often strongly interactive and together create an environmental envelope within which insect species survive and reproduce. Hodkinson (2005) further emphasizes that the abovementioned parameters combine to produce a general decrease in the overall structural complexity of the insects' habitat with increasing altitude.

According to Hegde et al. (2000a), the growth and size of a population depends on several environmental factors in addition to genetic structure. In the present study, consideration of the common and abundant species shows that numerical variation exists in regard to these species at all five altitudes. The occurrence of the dominance of one species over the others in any given area can be correlated with the dominant species' ecological versatility to exploit the conditions available in those habitats. The present study corroborates with the work of Muniyappa and Reddy (1981), Hegde et al. (2001), and Vasudev et al. (2001). There may be many other unknown microclimatic conditions that could also affect the density of *Drosophila.* The results of our study are in concurrence with the work of Cooper and Dobzhansky (1956), Reddy and Krishnamurthy (1977), Hegde et al. (2001), all of which have shown the influence of microclimatic conditions on the diversity of *Drosophila*. The present findings are also in agreement with the work of Cooper and Dobzhansky (1956) on species of *Drosophila* inhabiting the Sierra Nevada Mountains of the Yosemite region of California, where some of the species occurred at all elevations at which collections were made (259-3353 m a.s.l.). The results of our study are also in agreement with the work of Guruprasad et al. (2010), who showed that the number and density of *Drosophila* species decreased with increasing altitude at Chamundi Hill in Mysore, Karnataka. In our study, the presence of more species at lower altitudes can be attributed to the existence of thick vegetation, which provided good sources of food, and a more congenial environment at lower altitudes than at the higher altitudes.

Significant variations in the density of *Drosophila* were noticed during different seasons of the year on Mount Japfu. The density was highest during monsoon season at all altitudes and lowest during the pre-monsoon season. Possible reasons for the high density during monsoon season could be the availability of adequate food in the form of rotting fruits and the congenial climate for multiplication of the flies. The fact that the fruiting season of many plants in the area coincides with the monsoon season offers support for this conclusion. The monsoon season is characterized by heavy rains, reductions in temperature, and increases in humidity. As the monsoon season recedes, rainfall and humidity decrease, leading to a dry climate. The population density also starts declining in post-monsoon season, reaching its minimum during the pre-monsoon season. Thus, the fluctuations in population size of *Drosophila* could be closely related to the wet and dry seasons. However, in temperate regions, the population density declines to an extremely low level during cold winter months, indicating the influence of temperature on the regulation of population size, as is the case in several *Drosophila* species inhabiting temperate regions (Dobzhansky 1943; Patterson and Wagur 1943; Dobzhansky and Pavan 1950; Williams and Miller 1952; Wakahama 1961).

According to the constant, accessory, and accident species, as well as the cluster analysis, our study indicates several species that coexisted had similar ecological preferences.

In Simpson's diversity index (D), 0 represents infinite diversity, and 1 represents no diversity, i.e., the greater the value of D, the lower the diversity. Applying this index to understand the measures of biodiversity of flies at different altitudes of Mount Japfu shows that the second lowest altitude studied (1800 m a.s.l.) had the lowest D-value, indicating more biodiversity compared to other altitudes. Hodkinson (2005) suggested that the altitudinal distribution of an insect species is controlled by its environmental tolerances, with maximum population size being achieved at some optimum elevation and population density declining with altitude above and below the optimum. The results of our study suggest that the optimum elevation on Mount Japfu for *Drosophila* diversity is at 1800 m a.s.l. From the eco-distributional analysis of *Drosophila* species on Mount Japfu, it is clear that the distributional pattern of a species or related group of species is uneven in space and time. The *Drosophila* community of Mount Japfu was highly diverse and depended on several environmental factors in addition to the genetic structure of the species present in it.
